# The power of multivariate approach in identifying EEG correlates of interlimb coupling

**DOI:** 10.3389/fnhum.2023.1256497

**Published:** 2023-10-13

**Authors:** Sophie Hascher, Anastasia Shuster, Roy Mukamel, Ori Ossmy

**Affiliations:** ^1^Centre for Brain and Cognitive Development, School of Psychological Sciences, Birkbeck, University of London, London, United Kingdom; ^2^Sagol School of Neuroscience and School of Psychological Sciences, Tel Aviv University, Tel Aviv, Israel

**Keywords:** EEG, interlimb coupling, multivariate analysis, bimanual coordination, artificial neural network

## Abstract

Interlimb coupling refers to the interaction between movements of one limb and movements of other limbs. Understanding mechanisms underlying this effect is important to real life because it reflects the level of interdependence between the limbs that plays a role in daily activities including tool use, cooking, or playing musical instruments. Interlimb coupling involves multiple brain regions working together, including coordination of neural activity in sensory and motor regions across the two hemispheres. Traditional neuroscience research took a univariate approach to identify neural features that correspond to behavioural coupling measures. Yet, this approach reduces the complexity of the neural activity during interlimb tasks to one value. In this brief research report, we argue that identifying neural correlates of interlimb coupling would benefit from a multivariate approach in which full patterns from multiple sources are used to predict behavioural coupling. We demonstrate the feasibility of this approach in an exploratory EEG study where participants (*n* = 10) completed 240 trials of a well-established drawing paradigm that involves interlimb coupling. Using artificial neural network (ANN), we show that multivariate representation of the EEG signal significantly captures the interlimb coupling during bimanual drawing whereas univariate analyses failed to identify such correlates. Our findings demonstrate that analysing distributed patterns of multiple EEG channels is more sensitive than single-value techniques in uncovering subtle differences between multiple neural signals. Using such techniques can improve identification of neural correlates of complex motor behaviours.

## Introduction

Interlimb coupling—where the movement of one limb interacts with the movement of another limb—is a fundamental aspect of human motor behaviour. It plays a crucial role in most activities, from daily tasks such as walking or typing on a keyboard to specialised actions like playing a musical instrument or driving a manual transmission car. This interference can occur due to neural, biomechanical, or task-related factors, leading to coordinated or compensatory adjustments in the movement patterns of multiple limbs to maintain stability or optimise performance.

Over decades, understanding the neural mechanisms underlying human interlimb coupling is of great interest in the field of neuroscience as it unravels fundamental principles of motor control and holds significant implications for motor learning (Swinnen et al., 1993, 1997; Walter, 1998) and the development of interventions for individuals with motor impairments (Garbarini and Pia, 2013; Pandian and Arya, 2014; Daneault et al., 2015; Schambra et al., 2019; Doost et al., 2021).

Previous research has made substantial progress in investigating the neural underpinning of human interlimb coupling using various neuroimaging techniques, including functional magnetic resonance imaging (Debaere et al., 2001; Maki et al., 2008; Dietz et al., 2015; Garbarini et al., 2015), positron emission tomography (Goerres et al., 1998), transcranial magnetic stimulation (Serrien et al., 2002; Steyvers et al., 2003), and electroencephalography (EEG; Gerloff and Andres, 2002; Rudisch et al., 2023). Among these techniques, EEG has the greatest advantage in examining neural dynamics associated with interlimb coupling. In this brief research report, we highlight the power of using a multivariate approach to identify neural correlates of interlimb coupling and demonstrate its benefits in an exploratory EEG study.

### Univariate approach to the neural underpinning of interlimb coupling

**Figure 1:**
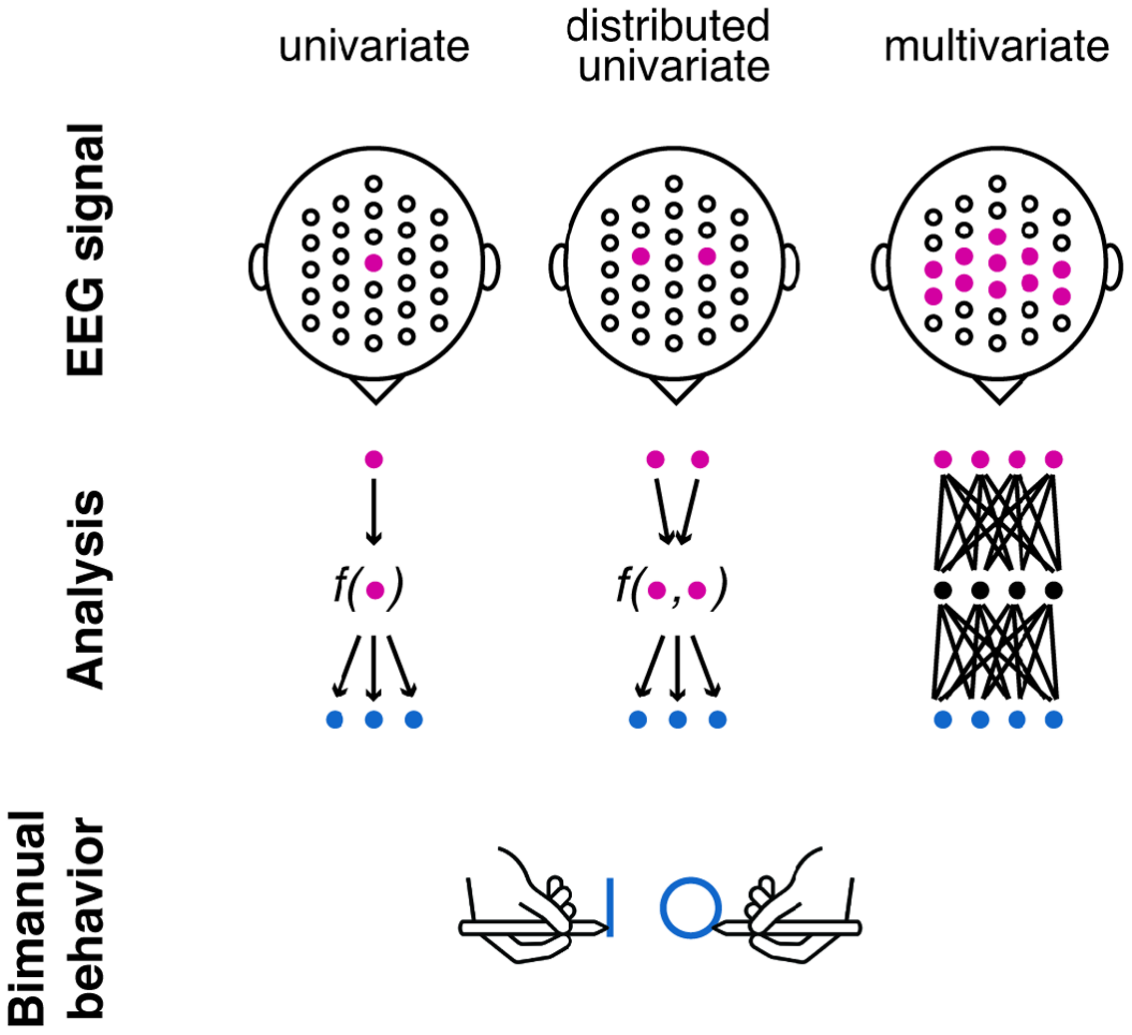
Different approaches for identifying neural correlates of interlimb coupling. Left panel: univariate approach focuses on one channel to predict the coupling effect (e.g., during bimanual behaviour when one hand draws a shape and another hand draws a different shape); Middle panel: distributed univariate approach takes into account multiple channels but reduce it to one value that predict the coupling effect; Right panel: multivariate approach examines non-linear relations between the EEG signal and the coupling effect. The blue dots in this schema indicate predicted behavioural outcomes. In our exploratory study, we used one behavioural outcome—CI value.

Traditional neuroscience research takes a univariate approach when investigating the neural underpinning of interlimb coupling. This approach involves examining individual features of EEG signals such as amplitude, latency, or phase at specific electrode sites or time points (Serrien et al., 2012; Kourtis et al., 2014), to identify neural activity that is associated with a particular interlimb movement (that is, interaction between different limbs or body parts as observed in walking, running, throwing, or climbing; Figure 1, left panel). This approach provides quantifiable, precise, and localised information about the EEG activity during interlimb-coupling tasks (Serrien et al., 2003, 2012; Kourtis et al., 2014; Desrochers et al., 2020) including bilateral symmetrical tasks (both hands perform in-phase synchronous movements), bilateral asymmetrical tasks (each limb performs different movements simultaneously), and interlimb coordination tasks (performing movements that involve alternating or simultaneous actions between the limbs as tapping, walking, or tracing patterns). One prominent univariate measure is event-related desynchronisation (ERD)—a reduction in oscillation power at alpha or beta frequencies. Univariate analysis shows differences in this measure between in-phase and out-of-phase movements (Deiber et al., 2005; Li et al., 2005).

### Distributed univariate EEG analysis

Univariate EEG analyses provide valuable insights into the involvement of specific cortical areas during interlimb coupling. Yet, these analyses neglect the holistic nature of interlimb motor control and are limited in capturing the interdependencies between brain regions across both hemispheres. To overcome this limitation, distributed univariate EEG techniques have emerged as a comprehensive framework for studying the neural correlates of motor behaviour (Figure 1, middle panel). Such analyses, instead of examining neural features from a single EEG channel, a single feature of coordinated activity across multiple brain regions is extracted (Kang and Druckmann, 2020).

A powerful distributed univariate EEG approach is functional connectivity analysis. Functionally connected regions exhibit synchronised features of neural activity, reflecting their involvement in shared motor processes (Lu et al., 2011; Demuru et al., 2013; Hoshino et al., 2021). There is a variety of techniques to examine functional connectivity in motor control using EEG signals (Babiloni et al., 2005; Tallet et al., 2009; Li et al., 2015). Most common technique is cross-channel coherence which quantifies the extent of similarity in neural activity between pairs of channels across various frequency bands (Tomiak et al., 2017; Weersink et al., 2019) and thereby sheds light on how regions communicate to allow interlimb control (Lu et al., 2011; Depestele et al., 2023). Previous work shows that coherence between channels across hemispheres over primary sensorimotor and premotor areas (C1 and C3 with C2 and C4) and midline (FCz or Cz) within the alpha and beta frequency band (8-13 Hz and 13–30 Hz respectively) subserves interlimb task execution and coupling (Serrien and Brown, 2004; Van Hoornweder et al., 2022). Others showed enhanced phase synchronisation (as a measure for cross-channel relations) between bilateral primary motor cortex and premotor cortex during interlimb coupling (Li et al., 2015). This phase synchronisation varies depending on the specific task characteristics (Andres et al., 1999; Gerloff and Andres, 2002).

### Multivariate EEG analysis: a comprehensive framework

Most interlimb movements are continuous and span relatively long time windows. Therefore, they rely on multiple temporal and spatial relationships between neural signals. Because univariate or distributed univariate approaches focus on isolated measures, they may fail to capture this complexity. Moreover, univariate approaches rely on linear assumptions or simplistic linear transformations in the signal and therefore cannot model non-linear relationships between the EEG signal and interlimb coupling, and neglect multidimensional patterns within the data. For these reasons, univariate approaches are limited in capturing subtle changes in neural activity related to interlimb coupling behaviours, hindering a comprehensive examination of the phenomenon (Groppe et al., 2011a,b).

In addition, univariate approaches typically involve averaging across participants or trials to derive group-level results (Wiesendanger and Serrien, 2004; Rueda-Delgado et al., 2014). The reliance on averaging poses challenges when studying personalised behavioural changes occurring over relatively long time periods—changes due to learning, development, injury, and rehabilitation. Previous research that examined individual differences in motor behaviour utilised a multivariate approach (Figure 1, right panel) alongside univariate approaches. For example, graph-theory measures capture network properties underlying functional connectivity in motor control (Fallani et al., 2010; Ismail and Karwowski, 2020). These measures represent brain regions as nodes and functional connections as edges, constructing a network that characterises the organisational aspects and efficiency of information flow among motor-related brain regions at the individual level. Such holistic approach is well-suited to capture the interdependencies and interactions between neural signals underlying movements from multiple limbs simultaneously.

Machine learning-based approaches, such as pattern recognition algorithms and classification models (Bishop, 1995; Bishop and Nasrabadi, 2006), have also emerged as valuable tools for decoding the complex neural patterns during motor behaviours (Presacco et al., 2012; Bashar et al., 2016; Sadiq et al., 2019; Tortora et al., 2020; Tidare et al., 2021). By training these models on multivariate EEG data, researchers can develop robust classifiers that accurately differentiate interlimb movements or predict task performance based on neural patterns. For example, artificial neural networks (ANN; Krogh, 2008; Kurkin et al., 2018) are computational models inspired by biological neural networks. They differ in the types of processing elements (i.e., how each neuron processes information) and the weighted link between them (i.e., the specific manner in which the neurons are connected; Bishop, 1995; Hagan et al., 1997). Different structures and weights represent alternative mechanisms and may distinguish the underlying neural mechanisms between individuals. Taken together, the multivariate approach is a more sensitive technique that has the potential to depict nuances in the EEG data that cannot be captured using a univariate approach.

### Exploratory study

We tested the feasibility of applying a multivariate approach to identify neural patterns underlying behaviour and its potential benefit over univariate approaches. To that end, we used bimanual coupling as a model system for interlimb coupling and tested it using an influential paradigm—the circle-line coupling task (Franz et al., 1991). This task involves drawing lines or circles with both hands (congruent condition) or alternatively drawing lines with one hand while simultaneously drawing circles with the other (incongruent condition; Franz et al., 1991; Garbarini and Pia, 2013). This task is particularly effective in revealing the reciprocal influence of hand actions due to the observed tendency of participants to produce curved lines and line-like circles in the incongruent condition compared to the congruent one. Notably, the paradigm can also be extended to drawing tasks involving more complex and discontinuous shapes, such as combining squares with circles, thus further illuminating the constraints and characteristics of bimanual coupling (Franz, 2003).

We aimed to identify neural correlates of bimanual coupling using univariate and multivariate approaches. We hypothesised that employing a multivariate analysis (using ANN with multiple EEG features as input to the network) would yield superior results in comparison to a univariate approach (using a single EEG feature—ERDs from single channel and frequency band) and distributed univariate approach (using cross-channel coherence). Specifically, because the multivariate analysis holistically considers the composite influence of various features from distinct channels and spectral bands, we hypothesised that it would show better predictions of the level of interaction between the hands at the trial level compared to both univariate analyses.

To test this hypothesis, we used a digital version of the paradigm where participants drew with digital pens on a touch screen. The touch screen allowed fine-grained, pixel-level quantification of participants’ movement patterns with better temporal and spatial accuracy than drawing on paper. The touch screen registered each time the pens touched the screen thus allowing us to track the exact movement times with respect to the EEG signal.

**Figure 2:**
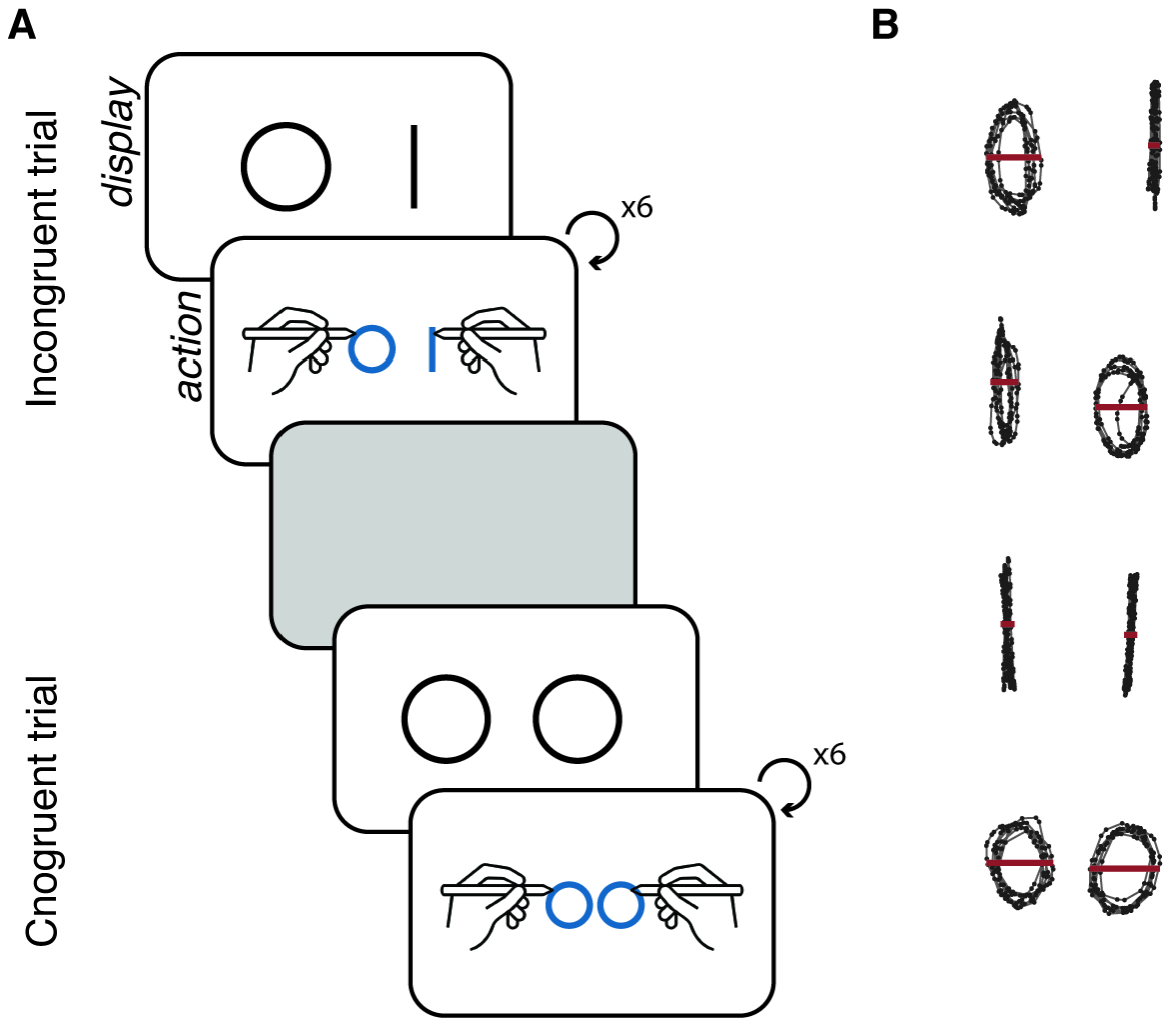
**(A)** Experimental design. Schematic illustration of two trials. Each trial started with a display on the screen of the condition-specific shapes, indicating the shapes that participants were required to draw. Upon initiating pen movement, the stimuli disappeared, and participants were able to view their own ongoing drawing. Each trial concluded after participants completed six iterations of the given shape with each hand. Between trials, participants were given a rest period of 5 s, during which they were instructed to hold the pens without any movement. **(B)** Behavioural measure. Example of the curvature-based measure for each experimental condition. In the incongruent conditions, the shapes became more similar to each other compared to the congruent conditions—the line drawing is more circle-shaped and the circle is more line-shaped. For each shape, we measured the distance of the right-most and left-most edges of the shape on the horizontal axis (red line; C_t_).

Behaviourally, we calculated the curvature of each one of the two drawn shapes (Figure 2). The change in curvature between the congruent and incongruent conditions reflects the mutual influence of the hands on each other and provides a direct link to the underlying motor control processes involved in the bimanual coupling effect. We then examined whether changes in curvature across drawing trials correlated with ERD in motor-related channels (univariate approach), coherence between motor-related channels (distributed univariate approach), and patterns detected from all motor-related channels using ANN (multivariate approach).

## Methods

### Participants

Ten right-handed healthy volunteers participated in the EEG study (7 females, mean age 22.04 years, range 20–29 years). All participants had normal or corrected-to-normal vision and provided written informed consent to participate in the study. The study conformed to the guidelines approved by the ethics committee at Tel-Aviv University.

### Task and design

Participants performed the circles-lines drawing task (Franz et al., 1991) in which they simultaneously drew either a line or a circle with each of their hands. We had 4 conditions: (1) line-line—participants had to draw a line with each hand; (2) circle-circle—participants had to draw a circle with each hand; (3) line-circle—participants had to draw a line with their left hand and circle with right hand; and (4) circle-line—participants had to draw a circle with their left hand and a line with their right hand.

We used a digital tablet (iPad 2, iOS6, Apple Inc.) to present participants with the shapes they needed to draw in each trial and gave them two compatible stylus digital pens (Adonit Jot Pro) for performing the drawing itself. The pens featured a precision disc tip that allowed for accurate and fine-tuned drawing on the tablet screen. Once participants touched the tablet with their pens, the shapes stimuli disappeared and the participants drew the two shapes (each with one hand) repeatedly six times. Participants received visual feedback of their drawing (the trace of their drawing appeared on the screen). Participants were instructed to try as much as possible to draw the shape in the image at the beginning of the trial.

There were 240 trials (60 per condition in random order; see Figure 2A for experimental design) in pseudo-random order (no condition repeated more than 4 consecutive times). The trials were separated by 5 s of rest where participants were asked to hold the pens and not move. Then, the condition was presented to them on the screen (the shapes they need to draw) and they were asked to start drawing. Once they start moving the pen, our stimulus was removed and they saw their own drawing. The trial ended after the participant completed 6 iterations of the shape in each hand. To ensure that participants understood the task, they had initial practice with the task where they performed 5–6 trials until they felt comfortable with the instructions and they were ready to begin. The experiment was custom-built and programmed in Apple XCode.

### Behavioural analysis: curvature calculation

For each shape, we measured the distance of the right-most and left-most edges of the shape on the horizontal axis (see Figure 2B), using the following formula:


$$C_{t\_\mathrm{left}}=|C_{x\_\mathrm{left}\_\max}-C_{x\_\mathrm{left}\_\min}|$$

$$C_{t\_\mathrm{right}}=|C_{x\_\mathrm{right}\_\max}-C_{x\_\mathrm{right}\_\min}|$$

Where C_t_left_ corresponds to the curvature of the shape that was drawn by the left hand in trial *t* and C_t_right_ corresponds to the curvature of the shape that was drawn by the right hand in trial *t*.

We then used a curvature-based measure to assess the level of bimanual coupling in each trial. High level of coupling in incongruent trials indicates strong interference of one hand with the second hand. In congruent trials, high levels of coupling are expected and indicate joint movement. Our measure CI was calculated according to the following formula:


$$CI=\frac{1}{\left|C_{t\_\mathrm{left}}-C_{t\_\mathrm{right}}\right|}$$

$CI_{t}$ serves as an objective and quantifiable measure of the degree of bimanual coupling effect in trial *t*. The greater its value, the smaller the difference in left and right curvature and therefore the stronger the coupling effect between the hands.

Previous research examined the possible influence of spatial constraints in the coordination and control of interlimb coupling in the circle-line task (Franz et al., 1991; Garbarini and Pia, 2013). These studies showed that when participants are asked to produce two movements of different spatial forms—circles and lines—they tend to exhibit spatial accommodation in the performances of both shapes, and this was manifested in a tendency for each shape to look more like the shape being performed by the other hand. According to these papers, this increase in spatial similarity between the shapes represents interlimb coupling. The quantification of this spatial similarity hinges on the calculation of curvature disparities between the shapes, encapsulating intricate geometrical differentiations. The CI value is an assessment of the discrepancy in shapes which allows for a rigorous assessment of shape alignment, facilitating a precise and objective evaluation of their structural resemblance.

Our analyses focused on individual participants and therefore the CI values served as our behavioural measure which we correlated with several neural features, per participant and across trials. We did not correlate across participants. CI values are expected to be high in congruent trials (movements are similar) compared to incongruent trials (movements are different).

### EEG data acquisition and preprocessing

The EEG analog signal was recorded continuously via 64 Ag–AgCl pin-type active channels mounted on an elastic cap according to the extended 10–20 method of electrode placing (BiosemiTM Active II system,^1^ Amsterdam, Netherlands). Seven additional channels were used: two mastoid channels (right and left), one electrode on the tip of the nose and four EOG channels for eye movement monitoring (two placed at the outer canthus of each eye and two placed at the orbital ridge centered directly above and below the right eye). EEG was digitised at a sampling rate of 256 Hz.

Data was analysed offline using the EEGLAB tool for MATLAB (Delorme and Makeig, 2004; Delorme et al., 2007). Raw EEG data was Band-pass filtered offline between 0.5 and 35 Hz (Butterworth filter, 24 dB), and re-referenced offline to the digital average of the two mastoids. The continuous data were segmented into epochs. The epochs differ in length and were based on the length of each trial. Therefore they were from −2000 ms relative to trial onset until the trial offset. Eye movements and blinks were detected and removed using independent component analysis (ICA; Makeig et al., 2000; Delorme et al., 2007).

Event-related spectral dynamics were computed using a continuous Morlet wavelet transform. For each trial in each participant, we computed the logarithm of the power (from trial onset to trial offset) relative to power during baseline (from 2000 ms to 500 ms pre trial onset). This procedure resulted in 200 (time points) × 50 (frequencies) matrix per trial in which cells indicated the log ratio value in the specific time point and frequency. A negative log ratio indicates a suppression in the power of EEG oscillations relative to baseline, whereas positive log ratios indicate enhanced power.

### Univariate approach: ERD

Bimanual tasks lead to event-related deysnchronisation (ERD) in individual motor-related channels in both hemispheres (Gerloff and Andres, 2002). In our analyses, for the univariate approach, we calculated ERDs for each participant and trial in each frontal-central channel (FC3, FC4, FCz), central channel (C3, C4, Cz), and central-parietal channel (CP3, CP4, CPz) on the alpha band (8–13 Hz) and beta band (13–30 Hz). The selection of these channels and frequencies is based on previous research showing their relevant to bimanual movements (Gerloff and Andres, 2002; Matthews et al., 2009). For that, we averaged the log ratio values in the time-frequency matrix of each trial and each channel across all time points and frequencies that are within the relevant band. For each participant, we calculated the correlation between each of the ERD values and CI values across trials (total of 9 channels × 2 frequency bands = 18 different correlations).

### Distributed univariate approach: coherence analysis

As a distributed univariate approach, we adopted an established coherence analysis that showed correspondence between EEG oscillations from motor-related channels and bimanual coordination (Tomiak et al., 2017). We calculated coherence in the frontal-central group (average of coherences between three pairs of the frontal-central channels FC3–FC4, FC3–FCz, and FCz- FC4), central group (the average of coherences between three pairs of central channels: C3–C4, C3–Cz, and C4–Cz), and the central-parietal group (CP3–CP4, CP3–CPz, and CP4–CPz). These average coherence values were calculated for the alpha band and the beta band separately. In total, for each participant and each trial, we had 6 coherence values (3 channel groups × 2 frequency bands; For full details and equations of the coherence calculation, see Tomiak et al., 2017). For each participant, we calculated the correlation between each of the coherence values and the CI values across trials.

### Multivariate approach: artificial neural network

As a multivariate approach, we designed an analysis using artificial shallow neural network which is characterised by a solitary hidden layer. We selected this architecture, as opposed to more complex models like convolutional neural network, to provide simplicity and interpretability by having low number of free parameters and to mitigate overfitting risks by maintaining a succinct model structure (Yegnanarayana, 2009; Lamb et al., 2011).

The network architecture was implemented with MATLAB Deep Learning Toolbox using mean squared error as a performance function and Variable Learning Rate Backpropagation as training function. The network comprised one input layer with 3600 nodes (200 time points × 2 frequency bands × 9 channels), a hidden layer with 100 nodes (based on previous research with a similar size of input and output layers; Mahira et al., 2014; Xu et al., 2022), and one output layer with 1 node (CI value estimation). This is the main difference between the univariate and multivariate approaches. In the ERD analysis, continuous data from a single channel were transformed to a single measure. In the coherence analysis, continuous data from pairs of channels were transformed to a single measure. Here, using ANN, we took all the data into account. Importantly, the addition of input does not automatically imply better prediction of the behaviour because irrelevant input (e.g., noise) can impede performance and make it harder for the algorithm to converge on the informative features.

The following procedure was conducted: First, we averaged the log ratio values in the time-frequency matrix of each trial and each channel across the time-frequency matrix of each band (alpha and beta). Please note we kept the temporal dynamics by not averaging across time points. Then we created 3600 (log ratio values in each time point, frequency band and channel) × 240 (trials) matrix indicating the patterns of neural activity for each participant. Then, we randomly selected 204 trials (85%) as a training set, 12 trials (5%) as validation set, and the remaining 24 trials (10%) as testing set. For the testing set, the network provided estimated curvature values for each trial and participant.

This partitioning aims to ensure the robustness and reliability of the network. The training set, comprising most of the data, is used to adjust the internal parameters of the model to minimise the error between predicted and actual outputs. This training phase is crucial for enabling the model to recognise non-linear patterns. The validation set functions as an essential aid in controlling the training process and preventing overfitting. The performance of the model during training is evaluated based on the validation set, which is distinct from the training set, and therefore makes sure the model does not become too specific to the training data and performs poorly on unseen instances. The process of training is halted when the performance on the validation set starts to degrade (‘Early Stopping’ procedure; Bishop, 1995; Wang et al., 2007), which enhances the generalisation of the model. The test set serves as the ultimate ‘assessment benchmark’ for the trained model. This set remains untouched during the training and hyperparameter tuning stages, allowing for an unbiased evaluation of the model’s ability to generalise to entirely unseen data.

To assess network performance, we calculated the correlation between the estimated CI values and the real CI values using Pearson correlation. We performed 1000 iterations of this procedure and each time randomly split the data to train, validation, and test sets. For each iteration, we received *r*-value and calculated a final mean *r*-value across all iterations. We assessed the significance of our results based on the mean *r*-value. We verified that each trial appeared in at least 5 iterations.

Because the random split was performed across 1000 iterations, the neural network was trained and validated multiple times on different subsets of the data. This process averts overfitting, as the network’s generalisation performance is evaluated using a diverse range of validation sets. Because we considered the overall performance of the network across multiple random splits, we provide a reliable evaluation of the model’s effectiveness. This procedure adheres to establish the best practices and statistical principles, warranting robustness and accuracy in the analysis of the neural network’s results.

## Results

One participant was excluded from the analysis due to a technical issue with syncing the EEG computer with the tablet. All further analyses were conducted on the remaining 9 participants. The average trial duration across participants was M ± SD = 7.26 ± 1.91 s. A repeated-measures ANOVAs confirmed main effect of drawing condition *F*(3,531) = 60.03, *p* < 0.01. Sidak-corrected *post-hoc* tests showed that congruent conditions took less time to complete compared to incongruent conditions but it differed across participants. Some of them did not exhibit any difference in trial duration across conditions and some did.

**Figure 3:**
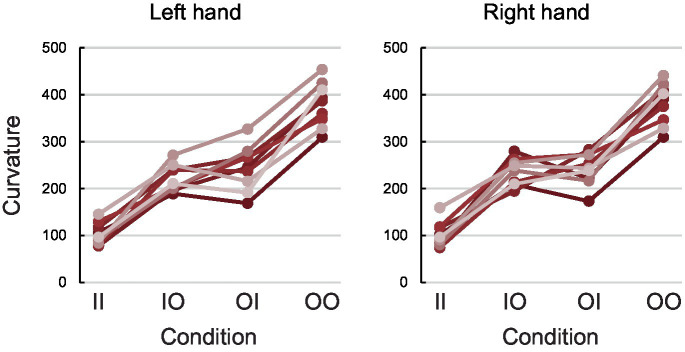
Behavioural results. Participants’ average Ct_left (left panel) and Ct_right (right panel) in pixels for each condition. Each colour denotes a different participant. The results verify the expected behaviour and replicate previous studies—in all participants and both hands, curvature was smallest in line-line condition, then incongruent conditions (circle-line and line-circle) and highest in circle-circle condition.

Figure 3 shows participants’ performance with their left and right hand (C_t_left_ and C_t_right_ respectively; see Methods) in each one of the experimental conditions (line-line, circle-line, line-circle, and circle-circle). Participants showed the expected performance which is similar to previous studies (Franz et al., 1991; Franz, 2003; Garbarini and Pia, 2013): the curvature for both hands was smallest in the line-line condition (Mean C_t_left_ ± SD = 103.08 ± 23.61 pixels; Mean C_t_right_ ± SD = 102.21 ± 26.49 pixels), then in the circle-line (Mean C_t_left_ ± SD = 222.13 ± 28.77 pixels; Mean C_t_right_ ± SD = 234.34 ± 28.99 pixels) and line-circle (Mean C_t_left_ ± SD = 244.14 ± 47.90 pixels; Mean C_t_right_ ± SD = 241.51 ± 34.34 pixels) conditions, and biggest in the circle-circle condition (Mean C_t_left_ ± SD = 379.28 ± 47.04 pixels; Mean C_t_right_ ± SD = 380.36 ± 44.30 pixels).

Nevertheless, to capture the bimanual coupling effect, we were interested in the modulation of one hand movement on the other hand movement and not in the mere performance of each one of the hands separately. Therefore, our main behavioural measure was CI (see Methods). For congruent trials, the coupling is high because both hands are performing the same action and CI values are expected to be high. For incongruent trials, high CI measures indicate strong influence of one hand over the other whereas low CI measures indicate weak coupling effect. We expected low CI values in incongruent trials. Indeed, participants exhibit higher average CI values in congruent trials compared to incongruent trials (M_CI_ ± SD = 0.09 ± 0.02 and 0.006 ± 0.002 for congruent and incongruent conditions respectively). Mixed repeated-measures ANOVA confirmed main between-subject effect *F*(3,531) > 109.49, *p* < 0.01, main effect of condition *F*(24,531) > 3.31, *p* < 0.01, and interaction *F*(8,531) > 3.76, *p* < 0.01. Sidak-corrected post-hoc tests confirmed that incongruent trials have lower CI value and that some participants were better in reducing the coupling effect than others.

At the neural level, we first examined the correlation between ERD at each motor-related channel and motor-related frequency bands (alpha: 8–12 Hz and beta: 15–30 Hz; see Methods) and the CI measure across trials per participant (total of 9 channels × 2 frequency bands × 9 participants = 162 correlations). In three participants there was at least one significant correlation *r*s(238) > 0.28, *p*s < 0.01 (uncorrected; Supplementary Table S1), but they were not consistent (no common channel across participants). Other correlations were not significant *p*s > 0.11.

Next, we took a distributed univariate approach by calculating cross-channel coherence as the neural measures (see Methods) and correlated it with the CI measure across trials per participant. Three of the nine participants had at least one significant correlation, overall there were 5 significant correlations *r*s(238) > 0.52, *p*s < 0.01 (uncorrected; Supplementary Table S2) whereas all other correlations were not significant *p*s > 0.19.

**Figure 4:**
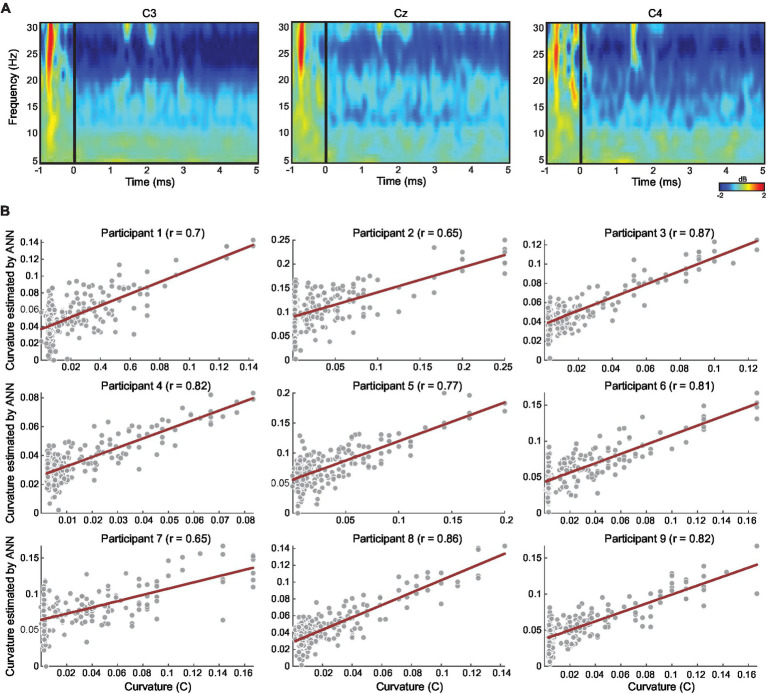
**(A)** Exemplar of event-related spectral perturbation map representing averaged changes in oscillation power across all trials for one participant (participant 7), locked to participant movement onset (time 0 ms) relative to baseline (−2000 to 0 ms). All trials were trimmed at 5 s only for this illustration, analyses were performed at the individual trial level. **(B)** Scatter plots per participant, showing correlation between the coupling measure CI (in all conditions; see Methods) and the CI value estimated by the ANN. The estimated CI value for each trial is the average of the estimated CI values across all the iterations in which the trial was in the test set (see Methods). Correlation was significant for all participants. The red line denotes the regression line.

Finally, we used ANN to estimate the CI measure based on neural activity recorded in the two motor-related frequencies from all motor-related channels (see Methods). We calculated the correlation between the predicted CI measure (ANN output, averaged across leave-one-out iterations; see Methods) and the actual CI measure across trials in each participant. Correlations in all 9 participants were significant *r*s(238) > 0.61, *p*s < 0.01. For each participant, Figure 4 shows the average of the CI values estimated by the ANN (across all the iterations) and the average correlation between the estimated values and the real CI values across all trials.

## Discussion

In this brief research report, we examined the use of univariate, distributed univariate, and multivariate approaches for identifying neural correlates of interlimb coupling. Using an artificial neural network (ANN) analysis, we were able to predict the level of interlimb coupling effect from EEG signal recorded from multiple channels simultaneously. In contrast, we did not find similar results using more traditional univariate approaches that focus on single channels (univariate) or coherence between channels (distributed univariate). Although correlates were found in some of the participants, they were not consistent in terms of channel or frequency band. Our findings provide evidence for the superiority of multivariate analyses in identifying neural correlates of interlimb coupling, demonstrating the potential of using this approach to expand existing literature in the field.

Our findings align with the growing recognition that the brain supports motor functions by operating a network with intricate interactions between distributed regions over time (Gerloff and Andres, 2002; Serrien and Brown, 2004; Chen X. et al., 2022; Chen Y.-F. et al., 2022). Some distributed univariate approaches such as cross-channel coherence capture functional connectivity between regions and therefore reflect this distributed-network perspective (Serrien et al., 2012; Kourtis et al., 2014), yet ANNs or other multivariate approaches are more powerful in extracting relevant features from the input data and reducing its dimensionality. This automatic extraction of salient features can capture important aspects of the data that may be missed by a distributed univariate approach.

Indeed, previous studies demonstrated how distributed patterns of EEG signals predict complex motor behaviours when using advanced machine learning algorithms (Ossmy et al., 2011; Bashar et al., 2016; Kurkin et al., 2018; Tortora et al., 2020; Hausmann et al., 2021), especially in atypical populations (Rad and Furlanello, 2016). For example, the use of multi-level classifiers and clustering analyses allowed for the identification of neural patterns and network-level dynamics involved in motor execution coordination (Schorer et al., 2007; Li et al., 2020), planning (Ieracitano et al., 2021; Mammone et al., 2021), and imagery (Amin et al., 2019). We contribute to this literature by showing that multivariate analysis tools can provide a more accurate and nuanced depiction of the neural mechanisms underlying interlimb coupling (Chen et al., 2022). Our findings further highlight the need to embrace multivariate approach as a valuable tool in studying behaviour. In future research, it is important to leverage the diverse structures and architectures of ANNs to explore alternative mechanisms for studying individual-level motor behaviour. Similar to our methodology, patterns of EEG signal during motor tasks can be used as input to the network and patterns of motor behaviours can be used as the output to predict. Comparison of the weights of each feature of the EEG signal and comparison of prediction accuracies between different ANN structures may reveal the contribution of each neural region to the observed movements.

The disparity between the successful identification of neural correlates using ANN and the failure to find such correlates using ERD or cross-channel coherence implies that linear models might not be adequate tools. Focusing on averaged, isolated measures overlook information about the nonlinearity between EEG activity and motor movements (Sadiq et al., 2019). Artificial neural networks, by their nature, excel in modelling nonlinear relationships, as they can capture complex patterns within the data.

In addition to averaging the activity in channels, traditional univariate approaches often rely on averaging activity across participants (Wiesendanger and Serrien, 2004; Rueda-Delgado et al., 2014). In contrast, we show that ANN allows the exploration of neural correlates at the individual level. ANNs are known for their ability to generalise patterns learned from training data to unseen data. This capability is highly advantageous when performing individual-level analyses because it allows to capture and model unique neural signatures and idiosyncrasies present in individual EEG data, enabling personalised analyses that go beyond group data. Examining neural correlates at the individual level holds paramount importance in motor neuroscience because it empowers researchers to uncover individual-specific patterns, better understand inter-individual variability in motor movement, and facilitate the exploration of personalised neural markers of motor dysfunction.

Finally, despite the advantages of multivariate approaches, their widespread adoption in studying motor behaviours has been relatively small. One of the main challenges is the computational complexity and technical requirements associated with multivariate analyses and their interpretability. Adequate training and collaboration between researchers with diverse skill sets, including neuroscientists, data scientists, and statisticians, are crucial to overcome these challenges. Promoting interdisciplinary collaborations and providing resources and training opportunities can facilitate the integration of multivariate approaches in the field of motor neuroscience.

## Data availability statement

The raw data supporting the conclusions of this article will be made available by the authors, without undue reservation.

## Ethics statement

The studies involving humans were approved by Tel Aviv University Ethics Committee. The studies were conducted in accordance with the local legislation and institutional requirements. The participants provided their written informed consent to participate in this study.

## Author contributions

SH: Formal analysis, Methodology, Validation, Writing – original draft, Writing – review & editing. AS: Conceptualization, Resources, Validation, Visualization, Writing – review & editing. RM: Conceptualization, Data curation, Funding acquisition, Investigation, Project administration, Resources, Supervision, Visualization, Writing – review & editing. OO: Conceptualization, Data curation, Formal analysis, Funding acquisition, Investigation, Methodology, Project administration, Resources, Software, Supervision, Validation, Visualization, Writing – original draft, Writing – review & editing.

## Footnotes

^1^ http://www.biosemi.com

## References

[ref1] Amin S. U. Alsulaiman M. Muhammad G. Mekhtiche M. A. Hossain M. S. (2019). Deep learning for EEG motor imagery classification based on multi-layer CNNs feature fusion. Futur. Gener. Comput. Syst. 101, 542–554. doi: 10.1016/j.future.2019.06.027

[ref2] Andres F. G. Mima T. Schulman A. E. Dichgans J. Hallett M. Gerloff C. (1999). Functional coupling of human cortical sensorimotor areas during bimanual skill acquisition. Brain 122, 855–870. doi: 10.1093/brain/122.5.855, PMID: 10355671

[ref3] Babiloni F. Cincotti F. Babiloni C. Carducci F. Mattia D. Astolfi L.. (2005). Estimation of the cortical functional connectivity with the multimodal integration of high-resolution EEG and fMRI data by directed transfer function. Neuroimage 24, 118–131. doi: 10.1016/j.neuroimage.2004.09.036, PMID: 15588603

[ref4] Bashar M.K. Chiaki I. Yoshida H. (2016). Human identification from brain EEG signals using advanced machine learning method EEG-based biometrics. Proceedings of the 2016 IEEE EMBS Conference on Biomedical Engineering and Sciences (IECBES). Kuala Lumpur, Malaysia: IEEE. 475–479.

[ref5] Bishop C.M. (1995). Neural networks for pattern recognition. New York: Oxford University Press.

[ref6] Bishop C.M. Nasrabadi N.M. (2006). Pattern recognition and machine learning. Berlin: Springer.

[ref7] Chen Y.-F. Fu R. Wu J. Song J. Ma R. Jiang Y.-C.. (2022). Continuous bimanual trajectory decoding of coordinated movement from EEG signals. IEEE J. Biomed. Health Inform. 26, 6012–6023. doi: 10.1109/JBHI.2022.3224506, PMID: 36423320

[ref8] Chen X. Li C. Liu A. McKeown M. J. Qian R. Wang Z. J. (2022). Toward open-world electroencephalogram decoding via deep learning: a comprehensive survey. IEEE Signal Process. Mag. 39, 117–134. doi: 10.1109/MSP.2021.3134629

[ref9] Daneault J.-F. Carignan B. Sadikot A. F. Duval C. (2015). Inter-limb coupling during diadochokinesis in Parkinson’s and Huntington’s disease. Neurosci. Res. 97, 60–68. doi: 10.1016/j.neures.2015.02.009 25747139

[ref10] Debaere F. Swinnen S. P. Béatse E. Sunaert S. Van Hecke P. Duysens J. (2001). Brain areas involved in interlimb coordination: a distributed network. Neuroimage 14, 947–958. doi: 10.1006/nimg.2001.0892 11697927

[ref11] Deiber M.-P. Ibañez V. Caldara R. Andrey C. Hauert C.-A. (2005). Programming effectors and coordination in bimanual in-phase mirror finger movements. Cogn. Brain Res. 23, 374–386. doi: 10.1016/j.cogbrainres.2004.11.009, PMID: 15820644

[ref12] Delorme A. Makeig S. (2004). EEGLAB: an open source toolbox for analysis of single-trial EEG dynamics including independent component analysis. J. Neurosci. Methods 134, 9–21. doi: 10.1016/j.jneumeth.2003.10.009, PMID: 15102499

[ref13] Delorme A. Sejnowski T. Makeig S. (2007). Enhanced detection of artifacts in EEG data using higher-order statistics and independent component analysis. Neuroimage 34, 1443–1449. doi: 10.1016/j.neuroimage.2006.11.004, PMID: 17188898 PMC2895624

[ref14] Demuru M. Fara F. Fraschini M. (2013). Brain network analysis of EEG functional connectivity during imagery hand movements. J. Integr. Neurosci. 12, 441–447. doi: 10.1142/S021963521350026X, PMID: 24372064

[ref15] Depestele S. van Dun K. Verstraelen S. Van Hoornweder S. Meesen R. (2023). Midfrontal theta and cognitive control during interlimb coordination across the adult lifespan. J. Mot. Behav. 55, 278–288. doi: 10.1080/00222895.2023.2183178, PMID: 36863697

[ref16] Desrochers P. C. Brunfeldt A. T. Kagerer F. A. (2020). Neurophysiological correlates of adaptation and interference during asymmetrical bimanual movements. Neuroscience 432, 30–43. doi: 10.1016/j.neuroscience.2020.01.044, PMID: 32036015

[ref17] Dietz V. Macauda G. Schrafl-Altermatt M. Wirz M. Kloter E. Michels L. (2015). Neural coupling of cooperative hand movements: a reflex and fMRI study. Cereb. Cortex 25, 948–958. doi: 10.1093/cercor/bht285, PMID: 24122137

[ref18] Doost M. Y. Herman B. Denis A. Sapin J. Galinski D. Riga A.. (2021). Bimanual motor skill learning and robotic assistance for chronic hemiparetic stroke: a randomized controlled trial. Neural Regen. Res. 16:1566. doi: 10.4103/1673-5374.301030 33433485 PMC8323667

[ref19] Fallani F. D. V. Costa L. D. F. Rodriguez F. A. Astolfi L. Vecchiato G. Toppi J.. (2010). A graph-theoretical approach in brain functional networks. Possible implications in EEG studies. Nonlinear Biomed. Phys. 4:S8. doi: 10.1186/1753-4631-4-S1-S8 20522269 PMC2880805

[ref20] Franz E. A. (2003). “Bimanual action representation: a window to human evolution” in Taking action: cognitive neuroscience perspectives on the problem of intentional acts. ed. Johnston-Frey S. (Cambridge, MIT Press), 259–288.

[ref21] Franz E. A. Zelaznik H. N. McCabe G. (1991). Spatial topological constraints in a bimanual task. Acta Psychol. 77, 137–151. doi: 10.1016/0001-6918(91)90028-X, PMID: 1759589

[ref22] Garbarini F. Pia L. (2013). Bimanual coupling paradigm as an effective tool to investigate productive behaviors in motor and body awareness impairments. Front. Hum. Neurosci. 7:737. doi: 10.3389/fnhum.2013.00737 24204339 PMC3817803

[ref23] Garbarini F. Turella L. Rabuffetti M. Cantagallo A. Piedimonte A. Fainardi E.. (2015). Bimanual non-congruent actions in motor neglect syndrome: a combined behavioral/fMRI study. Front. Hum. Neurosci. 9:541. doi: 10.3389/fnhum.2015.00541 26500520 PMC4594496

[ref24] Gerloff C. Andres F. G. (2002). Bimanual coordination and interhemispheric interaction. Acta Psychol. 110, 161–186. doi: 10.1016/S0001-6918(02)00032-X 12102104

[ref25] Goerres G. W. Samuel M. Jenkins I. H. Brooks D. J. (1998). Cerebral control of unimanual and bimanual movements: an H: 2: 15: O PET study. Neuroreport 9, 3631–3638. doi: 10.1097/00001756-199811160-00014 9858371

[ref26] Groppe D. M. Urbach T. P. Kutas M. (2011a). Mass univariate analysis of event-related brain potentials/fields I: a critical tutorial review. Psychophysiology 48, 1711–1725. doi: 10.1111/j.1469-8986.2011.01273.x, PMID: 21895683 PMC4060794

[ref27] Groppe D. M. Urbach T. P. Kutas M. (2011b). Mass univariate analysis of event-related brain potentials/fields II: simulation studies. Psychophysiology 48, 1726–1737. doi: 10.1111/j.1469-8986.2011.01272.x, PMID: 21895684 PMC4059014

[ref28] Hagan M.T. Demuth H.B. Beale M. (1997). Neural network design. Boston: PWS Publishing Co.

[ref29] Hausmann S. B. Vargas A. M. Mathis A. Mathis M. W. (2021). Measuring and modeling the motor system with machine learning. Curr. Opin. Neurobiol. 70, 11–23. doi: 10.1016/j.conb.2021.04.004 34116423

[ref30] Hoshino T. Oguchi K. Inoue K. Hoshino A. Hoshiyama M. (2021). Relationship between lower limb function and functional connectivity assessed by EEG among motor-related areas after stroke. Top. Stroke Rehabil. 28, 614–623. doi: 10.1080/10749357.2020.1864986, PMID: 33351724

[ref31] Ieracitano C. Morabito F. C. Hussain A. Mammone N. (2021). A hybrid-domain deep learning-based BCI for discriminating hand motion planning from EEG sources. Int. J. Neural Syst. 31:2150038. doi: 10.1142/S0129065721500386, PMID: 34376121

[ref32] Ismail L. E. Karwowski W. (2020). A graph theory-based modeling of functional brain connectivity based on eeg: a systematic review in the context of neuroergonomics. IEEE Access 8, 155103–155135. doi: 10.1109/ACCESS.2020.3018995

[ref33] Kang B. Druckmann S. (2020). Approaches to inferring multi-regional interactions from simultaneous population recordings. Curr. Opin. Neurobiol. 65, 108–119. doi: 10.1016/j.conb.2020.10.004, PMID: 33227602 PMC7853322

[ref34] Kourtis D. De Saedeleer L. Vingerhoets G. (2014). Handedness consistency influences bimanual coordination: a behavioural and electrophysiological investigation. Neuropsychologia 58, 81–87. doi: 10.1016/j.neuropsychologia.2014.04.002, PMID: 24732382

[ref35] Krogh A. (2008). What are artificial neural networks? Nat. Biotechnol. 26, 195–197. doi: 10.1038/nbt1386 18259176

[ref36] Kurkin S.A. Pitsik E.N. Musatov V.Y. Runnova A.E. Hramov A.E. (2018). Artificial neural networks as a tool for recognition of movements by electroencephalograms. In: ICINCO. 176–181. Available at: https://www.scitepress.org/papers/2018/68602/68602.pdf.

[ref37] Lamb P. F. Bartlett R. M. Robins A. (2011). Artificial neural networks for analyzing inter-limb coordination: the golf chip shot. Hum. Mov. Sci. 30, 1129–1143. doi: 10.1016/j.humov.2010.12.006, PMID: 21531031

[ref38] Li Y. Levin O. Forner-Cordero A. Swinnen S. P. (2005). Interactions between interlimb and intralimb coordination during the performance of bimanual multijoint movements. Exp. Brain Res. 163, 515–526. doi: 10.1007/s00221-004-2206-5, PMID: 15657696

[ref39] Li X. Mota B. Kondo T. Nasuto S. Hayashi Y. (2020). EEG dynamical network analysis method reveals the neural signature of visual-motor coordination. PLoS One 15:e0231767. doi: 10.1371/journal.pone.0231767, PMID: 32459820 PMC7252646

[ref40] Li W. Xu J. Chen X. He J. Huang Y. (2015). Phase synchronization between motor cortices during gait movement in patients with spinal cord injury. IEEE Trans. Neural Syst. Rehabil. Eng. 24, 151–157. doi: 10.1109/TNSRE.2015.2453311, PMID: 26208358

[ref41] Lu C.-F. Teng S. Hung C.-I. Tseng P.-J. Lin L.-T. Lee P.-L.. (2011). Reorganization of functional connectivity during the motor task using EEG time–frequency cross mutual information analysis. Clin. Neurophysiol. 122, 1569–1579. doi: 10.1016/j.clinph.2011.01.050, PMID: 21353633

[ref42] Mahira T. Imamoglu N. Gómez-Tames J. Kita K. Yu W. (2014). Modeling bimanual coordination using back propagation neural network and radial basis function network. Proceedings of the 2014 IEEE International Conference on Robotics and Biomimetics (ROBIO 2014). (pp. 1356–1361). Bali, Indonesia: IEEE.

[ref43] Makeig S. Enghoff S. Jung T.-P. Sejnowski T.J. Computation N. (2000). Moving-window ICA decomposition of EEG data reveals event-related changes in oscillatory brain activity. Proceedings of the 2nd International Workshop on Independent Component Analysis and Signal Separation, Helsinki. 627–632.

[ref44] Maki Y. Wong K. F. K. Sugiura M. Ozaki T. Sadato N. (2008). Asymmetric control mechanisms of bimanual coordination: an application of directed connectivity analysis to kinematic and functional MRI data. Neuroimage 42, 1295–1304. doi: 10.1016/j.neuroimage.2008.06.045, PMID: 18674627

[ref45] Mammone N. Ieracitano C. Morabito F.C. (2021). Mpnnet: a motion planning decoding convolutional neural network for EEG-based brain computer interfaces. Proceedings of the 2021 International Joint Conference on Neural Networks (IJCNN). Shenzhen:IEEE. 1–8.

[ref9002] Matthews A. J. Martin F. H. Garry M. Summers J. J. (2009). The behavioural and electrophysiological effects of visual task difficulty and bimanual coordination mode during dual-task performance. Exp. Brain Res. 198, 477–487. 19609513 10.1007/s00221-009-1943-x

[ref46] Ossmy O. Tam O. Puzis R. Rokach L. Inbar O. Elovici Y. (2011). Minddesktop-computer accessibility for severely handicapped. Proceedings of the International Conference on Enterprise Information Systems: SCITEPRESS. 316–320.

[ref47] Pandian S. Arya K. N. (2014). Stroke-related motor outcome measures: do they quantify the neurophysiological aspects of upper extremity recovery? J. Bodyw. Mov. Ther. 18, 412–423. doi: 10.1016/j.jbmt.2013.11.006, PMID: 25042312

[ref48] Presacco A. Forrester L. W. Contreras-Vidal J. L. (2012). Decoding intra-limb and inter-limb kinematics during treadmill walking from scalp electroencephalographic (EEG) signals. IEEE Trans. Neural Syst. Rehabil. Eng. 20, 212–219. doi: 10.1109/TNSRE.2012.2188304, PMID: 22438336 PMC3355189

[ref49] Rad N.M. Furlanello C. (2016). Applying deep learning to stereotypical motor movement detection in autism spectrum disorders. Proceedings of the 2016 IEEE 16th International Conference on Data Mining Workshops (ICDMW). Barcelona, Spain: IEEE. 1235–1242.

[ref50] Rudisch J. Fröhlich S. Pixa N. H. Kutz D. F. Voelcker-Rehage C. (2023). Bimanual coupling is associated with left frontocentral network activity in a task-specific way. Eur. J. Neurosci. 58, 2315–2338. doi: 10.1111/ejn.16042 37165733

[ref51] Rueda-Delgado L. M. Solesio-Jofre E. Serrien D. J. Mantini D. Daffertshofer A. Swinnen S. P. (2014). Understanding bimanual coordination across small time scales from an electrophysiological perspective. Neurosci. Biobehav. Rev. 47, 614–635. doi: 10.1016/j.neubiorev.2014.10.003, PMID: 25445184

[ref52] Sadiq M. T. Yu X. Yuan Z. Fan Z. Rehman A. U. Li G.. (2019). Motor imagery EEG signals classification based on mode amplitude and frequency components using empirical wavelet transform. IEEE Access 7, 127678–127692. doi: 10.1109/ACCESS.2019.2939623

[ref53] Schambra H. M. Xu J. Branscheidt M. Lindquist M. Uddin J. Steiner L.. (2019). Differential poststroke motor recovery in an arm versus hand muscle in the absence of motor evoked potentials. Neurorehabil. Neural Repair 33, 568–580. doi: 10.1177/1545968319850138, PMID: 31170880 PMC6631316

[ref54] Schorer J. Baker J. Fath F. Jaitner T. (2007). Identification of interindividual and intraindividual movement patterns in handball players of varying expertise levels. J. Mot. Behav. 39, 409–421. doi: 10.3200/JMBR.39.5.409-422, PMID: 17827117

[ref55] Serrien D. J. Brown P. (2004). Changes in functional coupling patterns during bimanual task performance. Neuroreport 15, 1387–1390. doi: 10.1097/01.wnr.0000131009.44068.51 15194858

[ref56] Serrien D. J. Cassidy M. J. Brown P. (2003). The importance of the dominant hemisphere in the organization of bimanual movements. Hum. Brain Mapp. 18, 296–305. doi: 10.1002/hbm.10086, PMID: 12632467 PMC6871910

[ref57] Serrien D. J. Sovijärvi-Spapé M. M. Farnsworth B. (2012). Bimanual control processes and the role of handedness. Neuropsychology 26, 802–807. doi: 10.1037/a0030154, PMID: 23106119

[ref58] Serrien D. J. Strens L. H. Oliviero A. Brown P. (2002). Repetitive transcranial magnetic stimulation of the supplementary motor area (SMA) degrades bimanual movement control in humans. Neurosci. Lett. 328, 89–92. doi: 10.1016/S0304-3940(02)00499-8, PMID: 12133562

[ref59] Steyvers M. Etoh S. Sauner D. Levin O. Siebner H. R. Swinnen S. P.. (2003). High-frequency transcranial magnetic stimulation of the supplementary motor area reduces bimanual coupling during anti-phase but not in-phase movements. Exp. Brain Res. 151, 309–317. doi: 10.1007/s00221-003-1490-9, PMID: 12756517

[ref60] Swinnen S. P. Lee T. D. Verschueren S. Serrien D. J. Bogaerds H. (1997). Interlimb coordination: learning and transfer under different feedback conditions. Hum. Mov. Sci. 16, 749–785. doi: 10.1016/S0167-9457(97)00020-1

[ref61] Swinnen S. P. Walter C. B. Lee T. D. Serrien D. J. (1993). Acquiring bimanual skills: contrasting forms of information feedback for interlimb decoupling. J. Exp. Psychol. Learn. Mem. Cogn. 19:1328. 8270889 10.1037//0278-7393.19.6.1328

[ref62] Tallet J. Barral J. Hauert C.-A. (2009). Electro-cortical correlates of motor inhibition: a comparison between selective and non-selective stop tasks. Brain Res. 1284, 68–76. doi: 10.1016/j.brainres.2009.05.058, PMID: 19497311

[ref63] Tidare J. Leon M. Astrand E. (2021). Time-resolved estimation of strength of motor imagery representation by multivariate EEG decoding. J. Neural Eng. 18:016026. doi: 10.1088/1741-2552/abd007, PMID: 33264756

[ref64] Tomiak T. Gorkovenko A. Mishchenko V. Vasilenko D. (2017). Features of EEG activity related to realization of cyclic unimanual and bimanual hand movements in humans. Neurophysiology 49, 78–89. doi: 10.1007/s11062-017-9632-z

[ref65] Tortora S. Ghidoni S. Chisari C. Micera S. Artoni F. (2020). Deep learning-based BCI for gait decoding from EEG with LSTM recurrent neural network. J. Neural Eng. 17:046011. doi: 10.1088/1741-2552/ab9842, PMID: 32480381

[ref66] Van Hoornweder S. Mora D. A. B. Depestele S. Frieske J. van Dun K. Cuypers K.. (2022). Age and interlimb coordination complexity modulate oscillatory spectral dynamics and large-scale functional connectivity. Neuroscience 496, 1–15. doi: 10.1016/j.neuroscience.2022.06.008, PMID: 35691515

[ref67] Walter C. (1998). Hot topics in motor control and learning: an alternative view of dynamical systems concepts in motor control and learning. Res. Q. Exerc. Sport 69, 326–333. doi: 10.1080/02701367.1998.10607706 9864750

[ref68] Wang W. Gelder P. H. V. Vrijling J. K. (2007). Comparing Bayesian regularization and cross-validated early-stopping for streamflow forecasting with ANN models. IAHS Publications Ser. Proc. Rep. 311, 216–221.

[ref69] Weersink J. B. Maurits N. M. de Jong B. M. (2019). EEG time-frequency analysis provides arguments for arm swing support in human gait control. Gait Posture 70, 71–78. doi: 10.1016/j.gaitpost.2019.02.017, PMID: 30826690

[ref70] Wiesendanger M. Serrien D. J. (2004). The quest to understand bimanual coordination. Prog. Brain Res. 143, 491–505. doi: 10.1016/S0079-6123(03)43046-X, PMID: 14653191

[ref71] Xu F. Xu X. Sun Y. Li J. Dong G. Wang Y.. (2022). A framework for motor imagery with LSTM neural network. Comput. Methods Prog. Biomed. 218:106692. doi: 10.1016/j.cmpb.2022.106692, PMID: 35248817

[ref72] Yegnanarayana B. (2009). Artificial neural networks. Delhi: PHI Learning Pvt Ltd.

