# Supplementary material for: The power of multivariate approach in identifying EEG correlates of interlimb coupling
Source: Front Hum Neurosci. 2023 Oct 13;17:1256497. doi: 10.3389/fnhum.2023.1256497 (PMC10603300; doi:10.3389/fnhum.2023.1256497)
Supplement: Supplementary file 1 [file Data_Sheet_1.PDF]

Supplementary Tables

**Participant**	**Band**	**Channel**	***r*(238)**	***p***
1	alpha	FC3	.39	<.01
1	alpha	FCz	.38	<.01
1	alpha	C3	.67	<.01
1	alpha	Cz	.73	<.01
3	alpha	C3	.28	<.01
3	alpha	C4	.44	<.01
3	beta	CPz	.36	<.01
6	beta	FC4	.49	<.01
6	beta	CP3	.43	<.01
6	beta	CP4	.39	<.01
6	beta	CPz	.41	<.01

Table S1. Significant Pearson correlations between average ERD and CI values (univariate approach)

**Participant**	**Band**	**Channel**	***r*(238)**	***p***
1	alpha	Frontal-Central	.52	<.01
1	alpha	Central	.61	<.01
5	alpha	Frontal-Central	.55	<.01
5	alpha	Central	.71	<.01
9	alpha	Central	.69.	<.01

Table S2. Significant Pearson correlations between cross-channel coherence and CI values (distributed univariate approach)
